# Effect of Periodontal Therapy on Crevicular Fluid Interleukin-6 and Interleukin-8 Levels in Chronic Periodontitis

**DOI:** 10.1155/2012/362905

**Published:** 2011-10-26

**Authors:** Paschalina Goutoudi, Evdoxia Diza, Malamatenia Arvanitidou

**Affiliations:** ^1^Department of Preventive Dentistry, Periodontology and Implant Biology, Dental School, Aristotle University of Thessaloniki, 70 Tsimiski Street, 54622 Thessaloniki, Greece; ^2^Department of Microbiology, Medical School, Aristotle University of Thessaloniki, 541 24 Thessaloniki, Greece; ^3^Department of Hygiene, Medical School, Aristotle University of Thessaloniki, 541 24 Thessaloniki, Greece

## Abstract

*Purpose*. The aim of this study was to analyse the levels of interleukin-6 (IL-6) and interleukin-8 (IL-8) in gingival crevicular fluid (GCF) of patients with chronic periodontitis prior to and following surgical and/or nonsurgical periodontal therapy for a period of 32 weeks. *Methods*. GCF samples were obtained from 24 nondiseased and 72 diseased sites of 12 periodontal patients prior to as well as at 6, 16, and 32 weeks following non-surgical and surgical periodontal therapy. IL-6 and IL-8 levels were determined by enzyme-linked immunosorbent assay (ELISA). *Results*. Periodontal treatment improved all clinical parameters. Both treatment modalities resulted in similar IL-6 as well as IL-8 levels. Mean IL-6 and IL-8 concentrations were significantly higher in non-diseased compared to diseased sites and increased significantly following treatment in diseased sites. Mean total amounts of IL-6 and IL-8 (TAIL-6, TAIL-8) did not differ significantly between diseased and nondiseased sites, while following therapy TAIL-8 levels decreased significantly. *Conclusions*. The data suggest that periodontal therapy reduced the levels of IL-8 in GCF. However, a strong relationship between IL-6, IL-8 amounts in GCF and periodontal destruction and inflammation was not found.

## 1. Introduction

Chronic periodontitis is an inflammatory disease affecting the supporting tissues of teeth. The expression of the disease results from the interaction of host defense mechanisms, microbial agents, environmental, and genetic factors. Various compounds, such as cytokines, have been detected in gingival crevicular fluid (GCF) [1] and may be especially beneficial for diagnosing current periodontal status and addressing the effects of periodontal treatment [2].

 IL-8 belongs to the interleukin-8 supergene family that includes small peptides with chemotactic activity for specific types of leukocyte populations [3]. This cytokine is induced and secreted by many cells, such as monocytes [4], lymphocytes [5], fibroblasts [6], epithelial, and endothelial cells [7, 8] as well as by synovial cells [6]. IL-8 attracts and activates polymorphonuclear leukocytes (PMN) in inflammatory regions [9, 10]. It induces the adhesion of PMN to endothelial cells and their transendothelial migration [11] as well as the release of granule enzymes from these cells [12].

 In periodontal patients, IL-8 has been reported in both GCF and periodontal tissues. McGee et al. [13] found that IL-8 concentrations were significantly higher in gingiva adjacent to probing pocket depth ≤3 mm and lowest adjacent to >6 mm sulci. In GCF, Chung et al. [14] suggested that the absence of a direct relationship between IL-8 and PMN recruitment may characterize individuals at risk for progression of periodontitis, while in another study [15], no significant difference in GCF IL-8 levels between localized juvenile periodontitis and healthy subjects was shown. According to Mathur et al. [16], the total amount of IL-8 was significantly higher in diseased compared to healthy sites.

Interleukin-6 (IL-6) is an important cytokine involved in the regulation of host response to tissue injury and infection [17]. It is produced by a variety of cells, such as monocytes, fibroblasts [18], osteoblasts [19], and vascular endothelial cells [20] in response to inflammatory challenges [21]. It plays an important role in B-cell differentiation [22] and in T-cell proliferation [23], while IL-6, synergistic with interleukin-1*β* (IL-1*β*), induces bone resorption [19]. However, it has also been reported that it can increase the production of tissue inhibitors of matrix metalloproteinases (TIMP) [24, 25], suppresses IL-1 expression [26], while it can induce the synthesis of IL-1 receptor antagonist (IL-1Ra) and the release of soluble TNF receptors [27] suggesting its anti-inflammatory properties.

In patients with refractory periodontitis, active sites—those displaying loss of attachment >2.1 mm in 3 months—revealed significantly higher GCF IL-6 levels than inactive ones [28]. In HIV-1-infected patients, GCF IL-6 levels were increased compared to uninfected periodontal patients [29]. According to Guillot et al. [30], in periodontal sites requiring surgery (unresolved sites), GCF IL-6 levels were significantly lower compared to those at resolved sites (not requiring surgery). In gingival tissues, Prabhu et al. [31] reported that expression of IL-6 m RNA was significantly higher in diseased tissues compared to healthy ones in periodontitis patients.

The purpose of this study was to examine the GCF levels of IL-6 and IL-8 in periodontal sites with varying degrees of destruction and inflammation of periodontal patients prior to and following surgical and/or nonsurgical periodontal therapy.

## 2. Materials and Methods

Patients with chronic periodontitis were recruited into this randomised, longitudinal, split-mouth, interventional study, from patients referred to the Department of Periodontology, Aristotle University of Thessaloniki. All of them were Caucasians. The selection criteria were (1) patients aged 35–65 years for males and 35–45 years for females; (2) good general health with no history of systemic disease; (3) no medication was taken; (4) no periodontal therapy received in the preceding 1 year; (5) more than 20 remaining teeth; (6) moderate to advanced periodontal disease as evidenced by multiple sites with a probing depth of 5 mm or more, extensive radiographic bone loss and bleeding on gentle probing; (7) pregnant or lactating females were excluded. Postmenopausal females or others on estrogen therapy were excluded [32]. Informed consent was obtained from each patient prior to enrolment in this study, and ethical approval was obtained from the Aristotle University of Thessaloniki ethics committee.

In each patient, two quadrants of either the mandible or maxilla were randomly assigned as experimental. In each experimental quadrant, 4 periodontal interproximal sites in single-rooted teeth were selected. Three sites displaying probing pocket depths (PD) ≥ 5 mm and a gingival index (GI) [33] of 2 or 3 were defined as diseased sites and 1 site with PD ≤ 3 mm and GI = 0 or 1 was defined as a nondiseased control site. A total of 96 test sites were included in the study, 72 of them as diseased and 24 as nondiseased sites. Sites in one experimental quadrant received nonsurgical periodontal treatment consisting of oral hygiene instructions, scaling and root surface debridement, while the contralateral sites received nonsurgical followed by surgical periodontal treatment, using a modified Widman flap [34]. At 6, 16, and 32 weeks following treatment, the dentition received supragingival polishing with a rubber cup and pumice.

Prior to as well as 6, 16, and 32 weeks following periodontal therapy, a GCF sample was taken from each test site, and IL-6 and IL-8 were quantified. The following clinical measurements were also evaluated: (1) plaque index (PlI), according to Silness and Löe [35], (2) gingival index (GI), according to Löe [33], (3) probing pocket depth (PD), and (4) clinical attachment loss (CAL), to the nearest millimeter with a Williams probe. The PD score in each site was evaluated in duplicate and mean values were, then, recorded. The same examiner performed all measurements. In all patients, individual acrylic stents were fabricated with reference grooves as reference points for the above clinical measurements and for GCF sampling.

Patients were all asked about their smoking habits and were classified as smokers or never-smokers. Former smokers, that is, patients who had stopped the habit, were not included in this study.

### 2.1. GCF Sampling

Both experimental quadrants were isolated with cotton rolls, and clinically detectable supragingival plaque was removed using a curette without touching the marginal gingiva. Sites were gently dried with an air syringe, and a single sterile paper strip (Periopaper, OraFlow, Plain View, NY, 11803, USA) for each examined site was inserted into the gingival crevice, until mild resistance was felt and was kept there for 30 s. Strips contaminated by bleeding were discarded and GCF sampling was repeated the following day. The amount of GCF collected was quantitated using Periotron 6000 (Siemens Medical Systems, Inc., Iselin, NJ, USA), which had been calibrated with 1 : 5 diluted serum [36]. Each paper strip was placed into a coded sealed plastic tube containing 250 *μ*L phosphate buffered saline (PBS). The samples were left at 4°C for 2 h and, then, they were frozen at −70°C and stored until cytokine analysis.

### 2.2. Enzyme-Linked Immunosorbent Assay (ELISA)

GCF was eluted from each filter paper strip into PBS as follows: before the IL-6 and IL-8 assays were performed, samples were left at 4°C for 2 h. Then, each strip was lifted to the surface of the eluent, and another 350 *μ*L of PBS was added to the strip (600 *μ*L final volume). Samples were, then, refrigerated at 4°C for another 20 min and centrifuged at 10,000 rpm for 10 min. Finally, the strips were discarded.

Commercial ELISA kits (R and D systems, Abingdon, Oxon, UK) were used to analyse IL-6 and IL-8 levels. The kit employs a quantitative “sandwich” enzyme immunoassay technique. A murine antihuman monoclonal antibody specific for IL-6 and IL-8 was precoated onto a 96-well microplate. Any IL-6 or IL-8 present was bound by the immobilised antibody. After washing of unbound proteins, an enzyme-linked (horseradish peroxidase) polyclonal antibody (200 *μ*L) specific for IL-6 or IL-8 (goat antihuman) was added to each well. Then, 200 *μ*L of a substrate solution was added and any colour developed was proportional to the amount of IL-6 or IL-8, respectively, bound in the initial step. The intensity of the colour (optical density) was measured using a microplate reader at 450 nm (wavelength correction set to 540 nm) within 30 min. A standard curve was prepared by plotting the concentration of the IL-6 (standards 300, 100, 50, 25, 12.5, 6.25, 3.12, and 0 pg/mL) or the concentration of the IL-8 standards (2000, 1000, 500, 250, 125, 62.5 31.2, and 0 pg/mL) against their optical density and the concentration of IL-6 or IL-8, respectively, was determined. Then, the pg of IL-6 or IL-8 in each sample (total amount) was calculated and the IL-6 or IL-8 concentration (pg/mL) was determined by dividing the amount of IL-6 or IL-8 by the GCF volume (*μ*L). The ELISA assays were run in duplicate; mean values were used to calculate total amounts and concentrations of each cytokine. The minimum detectable level of IL-6 (sensitivity of ELISA) was typically less than 0.70 pg/mL, while the minimum detectable level of IL-8 was less than 10 pg/mL.

### 2.3. Statistical Analysis

Data analysis was performed using the statistical package SPSS ver. 11.5 (SPSS Inc., Chicago Illsion, USA). For all time intervals, the mean cytokine and clinical values from the 2 healthy and the 6 diseased sites in each patient were used for the purposes of analysis. Negative samples were considered zero for the calculations. Differences in cytokine levels between diseased and nondiseased sites as well as between smokers and nonsmokers were evaluated by the Mann-Whitney test. In each case, the level of significance was set at *P* < 0.05. Comparison of the clinical measurements prior to and following therapy was performed by Wilcoxon Signed Ranks test. The same test was used to investigate the differences in GCF volume and in GCF IL-6 or IL-8 levels before as well as 6, 16, and 32 weeks following periodontal therapy. Finally, the Kendall's correlation coefficient was used to study the correlation between IL-6, IL-8 levels, and clinical parameters. A Bonferroni correction for all multiple comparisons was applied. To assess the distribution of the data, the Kolmogorov-Smirnov test was used.

## 3. Results

Twelve volunteers (7 females and 5 males, mean age 45.4 years) took part in this study. 

### 3.1. Frequency of Detection

Interleukin-6 was detected in GCF samples from 63/72 diseased sites (87.5%) and from 23/24 (95.83%) nondiseased sites, at baseline. The respective values at the 6th week following therapy were 60/72 (83.33%) and 22/24 (91.66%), at the 16th week 57/72 (79.17%) and 23/24 (95.83%), while at the 32nd week were 64/72 (88.89%) and 23/24 (95.83%).

At baseline, interleukin-8 was detected in GCF samples from 71/72 diseased sites (98.61%) and from all the nondiseased sites (100%). The respective values following therapy were: 71/72 (98.61%) and 23/24 (95.83%) at the 6th week, 70/72 (97.22%) and 23/24 (95.83%) at the 16th week, and, finally, 71/72 (98.61%) and 23/24 (95.83%) at the 32nd week.

### 3.2. GCF Volumes

The GCF volume, expressed in *μ*L, of 72 diseased and 24 nondiseased sites both prior to and following therapy are presented in Table 1. Mean GCF values were significantly higher in diseased compared to nondiseased sites (*P* < 0.01 at baseline, *P* < 0.05 following therapy). Periodontal therapy resulted in a significant decrease in GCF volume in both diseased and nondiseased sites (*P* < 0.01 and *P* < 0.05, resp.).

### 3.3. Concentration and Total Amount of IL-6 and IL-8

The concentration (IL-6, IL-8) and the total amount (TAIL-6, TAIL-8) of interleukin-6 and -8 in GCF expressed in pg/*μ*L and pg/30 s, respectively, are presented in Table 2. Mean IL-6 concentration values were significantly higher in nondiseased compared to diseased sites both prior to and following periodontal treatment (at baseline *P* < 0.01, post-treatment *P* < 0.05). Mean TAIL-6 values did not differ significantly between diseased and nondiseased sites. Periodontal treatment resulted in significant increase of IL-6 concentration in diseased sites (*P* < 0.01 at 6 weeks), while in nondiseased sites IL-6 levels remained almost unchanged. Following therapy, TAIL-6 levels were slightly reduced at 6 weeks, but at 32 weeks they were increased in almost pretreatment levels.

Mean IL-8 concentration was significantly higher in nondiseased compared to diseased sites (*P* < 0.01 at baseline and at 6 weeks). Following therapy, IL-8 concentration increased significantly (*P* < 0.05 at 6 and 16 weeks) in diseased sites, while in nondiseased it displayed only a slight increase. Mean TAIL-8 levels were similar in diseased and nondiseased sites. TAIL-8 decreased significantly following therapy (*P* < 0.01 at 6 weeks).

 Both treatment modalities, surgical and nonsurgical periodontal treatment, resulted in similar IL-6 as well as IL-8 total amounts and concentrations at each interval following treatment.

A strong correlation (*P* < 0.05) was also found between IL-6 and IL-8 concentrations (Table 3).

### 3.4. Clinical Parameters

In diseased sites, periodontal treatment led to improvements in all clinical parameters (Table 2). At 6 weeks, mean PD, CAL, PlI, and GI scores were significantly decreased (*P* < 0.01). The PlI and GI scores were also decreased (*P* < 0.05) at 16 weeks. However, by 32 weeks after-therapy an increase in PD, CAL, GI, and PlI scores was noted (*P* < 0.05 for GI and PlI). In nondiseased sites, periodontal therapy resulted in a significant decrease only in PlI and GI scores at 6 weeks (*P* < 0.01) and at 16 weeks (*P* < 0.05). At 32 weeks, an increase in PlI score was noted (*P* < 0.05).

The comparison between IL-6, IL-8 levels, and clinical parameters revealed strong negative correlations between IL-6 concentration and GI, PD and CAL (Table 4).

### 3.5. Smoking Status

The concentration and the total amount of IL-6 and IL-8 as well as the mean values of PD and CAL in seven smokers and five nonsmokers are presented in Table 5. In all sites, TAIL-6 levels were higher in nonsmokers than in smokers both prior to and following treatment (significantly in diseased sites at 6 weeks, *P* < 0.05). Similarly, IL-6 concentration was higher in nonsmokers compared to smokers without reaching statistical significance. 

At baseline, in all sites TAIL-8 levels were higher in nonsmokers (significantly *P* < 0.01 in nondiseased sites). Periodontal treatment led to reduced TAIL-8 levels in nonsmokers, while it did not seem to influence them in smokers. Following therapy, TAIL-8 levels were higher in smokers compared to nonsmokers (significantly at 32 weeks in diseased and at 6 weeks in nondiseased sites).

In diseased sites, IL-8 concentration in smokers increased following therapy, while it remained almost unchanged in nonsmokers. Thus, IL-8 was significantly higher in smokers post-treatment (*P* < 0.01). In nondiseased sites, IL-8 at baseline was higher in nonsmokers (*P* < 0.01), while following therapy IL-8 was higher in smokers (*P* < 0.01).

## 4. Discussion

The major pathophysiological role of interleukin-8 lies in affecting neutrophils [15, 37]. IL-8 levels in GCF, therefore, from patients during periodontal therapy could be helpful in monitoring the progression of periodontal disease. In our study, no clear differences in the total amount of IL-8 were observed, when diseased sites were compared with nondiseased ones implying either the inflammatory status of “healthy” sites or the role of IL-8 to the steady state of the gingival [38]. Additionally, the findings of previous studies [14, 39] suggested an inverse relationship between PMN recruitment responsible for the periodontal status and IL-8 levels in GCF. Mathur et al. [16], on the contrary, found that the total amount of IL-8 was higher in diseased compared to healthy sites. Periodontal treatment resulted in a significant decrease of mean TAIL-8 in diseased and nondiseased sites at 6 weeks. However, at that time, in 23 of the 72 diseased sites an increase of TAIL-8 was noted. Chung et al. [14] found that in some patients scaling and root planing led to decreased and in some others to increased levels of IL-8 and of *β*-glucuronidase, a PMN indicator, and tried to correlate them with individuals at risk for progression of periodontitis. In our study, the sites with increased IL-8 levels following therapy were not characterized by significant loss of attachment or inflammation. Weak correlations between TAIL-8 and clinical parameters were observed, positive with GI and negative with PD. This could be explained by the fact that clinical parameters, such as probing depth, clinical attachment loss, and bleeding on probing do not necessarily reflect current disease activity [38] as well as by the small number of patients and their heterogeneity. Both treatment modalities, surgical and nonsurgical, improved the clinical indices and resulted in lower TAIL-6 and TAIL-8 levels 6 weeks following therapy. At 32 weeks, these levels increased. At that time, however, the levels were similar in surgically and nonsurgically treated sites. These data suggest that in this split-mouth design research, where factors influencing the one half of the mouth might also influence the other half, the treatment modality did not influence the amounts of IL-6 and IL-8 in GCF 32 weeks following therapy.

 The concentrations of both IL-6 and IL-8 were significantly higher in nondiseased compared to diseased sites, while following periodontal treatment they increased significantly. These results could be due to the reduction of GCF volume following successful therapy. It has been suggested that in GCF the total cytokine amount might be more representative of the disease status as compared to the concentration [40]. According to Chapple et al. [41], GCF volumes are very variable irrespective of inflammatory status. Thus, it was proposed [41] that the total marker activity per 30 s GCF sample rather than the concentration of the marker might provide a better correlation with health or disease status.

 IL-6 has direct stimulatory effects on bone resorption [42], although this is controversial [43]. On the other hand, IL-6 was suggested to have anti-inflammatory properties [27]. In our study, neither disease severity nor inflammatory status seemed to influence significantly TAIL-6 levels. There was a weak negative correlation between TAIL-6 and PD or GI, while in nondiseased sites, mean TAIL-6 was numerically higher compared to diseased sites. Following treatment, TAIL-6 levels were slightly reduced at 6 and at 16 weeks. According to our findings, the total amounts of GCF IL-6 could hardly be correlated with either periodontal destruction or inflammation. In agreement with our results, Bozkurt et al. [44] suggested that in patients with adult periodontitis no correlation between GCF IL-6 levels and clinical parameters was found. However, previous results suggested a positive correlation of TAIL-6 with disease activity [45]. Geivelis et al. [46] found significant positive correlations between gingival bleeding as well as PD and IL-6. Atilla and Kütükçüler [47] detected higher GCF IL-6 levels in sites with gingivitis than in healthy ones, while Lee et al. [48] indicated higher GCF IL-6 levels in active than in inactive sites. The diversity of the results in different studies support the idea that the production of inflammatory mediators differs from site to site and from subject to subject and their levels may be influenced by several factors, such as genetic factors [49] and bacterial composition [50].

Smoking is an important environmental risk factor for the initiation and progression of periodontitis [51–53]. In agreement with previous findings [53], our results showed that smoking did not influence significantly IL-6 levels in GCF, although there was a trend for higher IL-6 levels in nonsmokers compared to smokers, significantly at 6 weeks following therapy. Before treatment, when the antigenic stimuli from the bacterial plaque in untreated periodontal pockets were high, the total amounts of IL-8 were higher in nonsmokers than in smokers. Following therapy, IL-8 levels decreased significantly in nonsmokers; on the contrary, they did not seem to be influenced in smokers. As a consequence, TAIL-8 levels were higher in smokers following treatment compared to nonsmokers, as it was suggested from a previous study on experimental gingivitis [54]. The concentration of IL-8 in nonsmokers remained almost unchanged following therapy, while in smokers it increased steadily and became significantly higher than in nonsmokers. Although one could consider the small number of patients studied, when interpreting the results, our observations suggest that smoking may keep the GCF content of IL-8 in high levels influencing the response to periodontal therapy. Additionally, in diseased sites a better clinical result was achieved in nonsmokers following treatment, as evident from the PD and CAL values. This is in agreement with previous observations that the outcome of periodontal therapy was significantly compromised in smokers [51, 52, 55].

## 5. Conclusions

Both treatment modalities improved significantly the clinical indicies; this improvement was accompanied by a down regulation of the mean total amount of IL-8 in GCF. The total amounts of IL-6, however, were not significantly influenced during the 32 weeks following therapy. A strong relationship between IL-6 or IL-8 levels in GCF and periodontal destruction or inflammation was not found. Moreover, the smoking status seemed to influence both the total amount and the concentration of IL-8 in diseased and nondiseased sites as well as IL-6 levels to a lesser extend. The value of monitoring GCF IL-6 and IL-8 was not evident in our study; further studies are required to clarify the exact role of these cytokines in periodontal disease and to evaluate other factors that in conjunction with local ones may influence their levels in GCF.

## Figures and Tables

**Table 1:** GCF volume (*μ*L) of diseased and nondiseased sites in 12 periodontal patients prior to and following therapy.

Periodontal sites	Baseline (mean ± SD)	6 weeks (mean ± SD)	16 weeks (mean ± SD)	32 weeks (mean ± SD)
Diseased	0.186 ± 0.042**	0.05 ± 0.013*	0.032 ± 0.014*	0.036 ± 0.013*
Nondiseased	0.033 ± 0.012	0.014 ± 0.005	0.016 ± 0.007	0.013 ± 0.006

*P < 0.05 between diseased and nondiseased sites.

**P < 0.01 between diseased and nondiseased sites.

**Table 2:** Concentration and total amount of IL-6 and IL-8 in GCF as well as PD, CAL, PlI, and GI prior to and following therapy in diseased and nondiseased sites of 12 periodontal patients.

Parameters	Baseline (Mean ± SD)	6 weeks (Mean ± SD)	16 weeks (Mean ± SD)	32 weeks (Mean ± SD)
IL-6 D	11.38 ± 4.73^∗∗##^	33.56 ± 10.54*	33.12 ± 12.86*	38.72 ± 11.77*
IL-6 ND	79.42 ± 34.21**	82.60 ± 37.52*	68.37 ± 18.12*	93.63 ± 24.04*
TAIL-6 D	1.06 ± 0.41	0.88 ± 0.37	0.79 ± 0.27	1.03 ± 0.27
TAIL-6 ND	1.39 ± 0.56	0.97 ± 0.35	0.99 ± 0.31	1.25 ± 0.39
IL-8 D	1103.76 ± 498.22^∗∗#^	2085.27 ± 664.01^∗∗#^	3243.4 ± 2271.12	3290.47 ± 609.77
IL-8 ND	3957.32 ± 1983.64**	5495.88 ± 1985.74**	4269.38 ± 2607.36	4692.78 ± 1267.21
TAIL-8 D	95.51 ± 40.03^##^	59.68 ± 24.78	62.63 ± 17.87	71.32 ± 14.64
TAIL-8 ND	101.97 ± 39.10^##^	58.12 ± 19.62	50.16 ± 10.37	54.96 ± 13.06
PD-D	6.10 ± 0.87^∗∗##^	3.83 ± 1.16**	3.46 ± 0.96**	3.57 ± 1.04**
PD-ND	2.42 ± 0.58**	2.17 ± 0.56**	2.29 ± 0.46**	2.29 ± 0.55**
CAL-D	7.35 ± 1.68^∗∗##^	5.93 ± 1.92**	5.57 ± 1.93**	5.755 ± 1.80**
CAL-ND	3.42 ± 1.28**	3.58 ± 1.18**	3.75 ± 1.11**	3.58 ± 1.14**
PlI-D	2.07 ± 0.51^∗∗##^	0.71 ± 0.46^∗∗#^	0.26 ± 0.44^#^	0.57 ± 0.53**
PlI-ND	1.21 ± 0.41^∗∗##^	0.33 ± 0.48^∗∗#^	0.08 ± 0.28^#^	0.29 ± 0.46**
GI-D	2.08 ± 0.28^∗∗##^	0.67 ± 0.58^∗∗#^	0.11 ± 0.32^#^	0.44 ± 0.50**
GI-ND	1.00 ± 0.00^∗∗##^	0.17 ± 0.38^∗∗#^	0.00 ± 0.00	0.04 ± 0.20**

D: diseased sites; ND: nondiseased sites; IL-6, IL-8: concentration (pg/*μ*L); TAIL-6, TAIL-8: total amount (pg/30 s).

*P < 0.05 between diseased and nondiseased sites.

**P < 0.01 between diseased and nondiseased sites.

^#^ *P* < 0.05 between two examination intervals.

^##^ *P* < 0.01 between two examination intervals.

**Table 3:** Correlation between total amount and concentration of IL-6 and IL-8.

	IL-6	TAIL-6	IL-8	TAIL-8
IL-6	1.000			
TAIL-6	0.412	1.000		
IL-8	0.385*	0.164	1.000	
TAIL-8	−0.189	0.237	0.439	1.000

*P < 0.05; IL-6, IL-8: concentration (pg/*μ*L); TAIL-6, TAIL-8: total amount (pg/30 s).

**Table 4:** Correlation between concentration, total amount of IL-6 and IL-8, and clinical parameters of 12 periodontal patients.

Parameters	IL-6	IL-8	TAIL-6	TAIL-8
PlI	−0.236	−0.212	0.095	0.209
GI	−0.323*	−0.278	−0.107	0.224
PD	−0.471*	−0.262	−0.279	−0.213
CAL	−0.385*	−0.121	−0.114	−0.185

*P < 0.05, IL-6, IL-8: concentration (pg/*μ*L), TAIL-6, TAIL-8: total amount (pg/30 s).

**Table 5:** Concentration and total amount of IL-6 and IL-8 in GCF as well as PD, CAL prior to and following therapy in diseased and nondiseased sites of 7 smokers and 5 nonsmoker periodontal patients.

Parameters	Baseline (Mean ± SD)	6 weeks (Mean ± SD)	16 weeks (Mean ± SD)	32 weeks (Mean ± SD)
*Diseased sites*				
IL-6 NS	15.85 ± 6.03	43.16 ± 11.86	32.50 ± 13.32	45.57 ± 12.44
IL-6 S	8.19 ± 2.35	26.70 ± 7.69	33.57 ± 11.67	33.82 ± 10.91
TAIL-6 NS	1.46 ± 0.57	1.38 ± 0.43*	1.12 ± 0.29	1.52 ± 0.39*
TAIL-6 S	0.78 ± 0.28	0.53 ± 0.20*	0.56 ± 0.22	0.68 ± 0.17*
IL-8 NS	1177.12 ± 496.41	1168.81 ± 509.83	1237.93 ± 337.84	1167.10 ± 305.47
IL-8 S	1051.36 ± 501.22^##^	2739.87 ± 823.54^##^	4675.88 ± 1062.09	4807.16 ± 938.66
TAIL-8 NS	118.89 ± 44.51	42.67 ± 17.18	40.66 ± 11.31	41.63 ± 9.87*
TAIL-8 S	78.80 ± 27.04	71.83 ± 30.45	78.32 ± 19.96	92.52 ± 20.94*
PD-NS	6.03 ± 0.81	3.67 ± 1.03	3.30 ± 0.79	3.27 ± 1.05*
PD-S	7.52 ± 1.85	3.95 ± 1.25	3.57 ± 1.06	3.79 ± 1.02*
CAL-NS	7.10 ± 1.40	5.53 ± 1.61	5.03 ± 1.63	5.13 ± 1.61*
CAL-S	7.52 ± 1.85	6.21 ± 2.08	5.95 ± 2.05	6.19 ± 1.81*

*Nondiseased sites*				
IL-6 NS	102.46 ± 41.36	92.23 ± 42.07	46.46 ± 12.53	106.38 ± 27.89
IL-6 S	62.97 ± 27.26	75.74 ± 17.89	84.03 ± 22.75	84.53 ± 20.11
TAIL-6 NS	1.93 ± 0.62	1.22 ± 0.41	1.20 ± 0.36	1.77 ± 0.48
TAIL-6 S	1.01 ± 0.41	0.79 ± 0.20	0.84 ± 0.18	0.88 ± 0.30
IL-8 NS	6010.00 ± 2028.23^∗∗##^	2656.83 ± 1001.99*	1624.08 ± 337.90**	2542.74 ± 679.54**
IL-8 S	2491.06 ± 793.85^∗∗##^	7523.77 ± 2898.40*	6158.88 ± 1095.12**	6228.52 ± 1809.06**
TAIL-8 NS	178.59 ± 53.09**	36.98 ± 11.87*	35.49 ± 8.06	41.89 ± 10.04
TAIL-8 S	47.24 ± 21.38**	73.20 ± 27.11*	60.64 ± 11.23	64.29 ± 17.43
PD-NS	2.30 ± 0.67	2.10 ± 0.32	2.00 ± 0.00*	2.00 ± 0.47*
PD-S	2.50 ± 0.52	2.21 ± 0.70	2.50 ± 0.52*	2.50 ± 0.52*
CAL-NS	3.20 ± 1.03	3.20 ± 1.03	3.40 ± 0.97	3.40 ± 0.84
CAL-S	3.57 ± 1.45	3.86 ± 1.23	4.00 ± 1.18	3.71 ± 1.33

NS: nonsmokers; S: smokers, IL-6, IL-8: concentration (pg/*μ*L); TAIL-6, TAIL-8: total amount (pg/30 s);

*P < 0.05 between smokers and nonsmokers.

**P < 0.01 between smokers and nonsmokers.

^##^ *P* < 0.01 between two examination intervals.
